# Dual Beneficial Effects of Topical l‐Glutamine on Oral Mucositis in 5‐Fluorouracil‐Treated Mice

**DOI:** 10.1111/jop.70135

**Published:** 2026-04-07

**Authors:** Juliana Francisca Grossi Heleno, Igor de Campos Fontes, Leticia Cristine Cardoso dos Santos, Victor Prokop Gonçalves Campos, Iury Berckmann Freitas Gomes, Derek William Gilroy, Silvia Passos Andrade, Paula Peixoto Campos

**Affiliations:** ^1^ Department of General Pathology, Biological Science Institute Federal University of Minas Gerais Belo Horizonte Brazil; ^2^ Department of Hospital Dentistry Benjamin Guimarães Foundation, Hospital of Baleia Belo Horizonte Brazil; ^3^ Department of Ageing, Rheumatology & Regenerative Medicine University College London London UK; ^4^ Department of Physiology and Biophysics Biological Science Institute, Federal University of Minas Gerais Belo Horizonte Brazil

**Keywords:** inflammation, l‐glutamine, remodeling, tongue lesions

## Abstract

**Background:**

Oral mucositis is a severe health and economic problem that occurs in about 40%–90% of patients undergoing cancer treatments. Systemic or topical application of l‐glutamine has been shown to improve mucosal healing and ameliorate oral mucositis (OM) symptoms according to clinical score systems. To further establish the beneficial effects of this amino acid, our study examined the actions of topical application of l‐glutamine on inflammation and mucosal healing markers in tongue lesions in mice treated with 5‐Fluorouracil.

**Methods:**

The animals received 3 doses of the chemotherapeutic agent (non‐consecutive days) at concentrations of 60, 40, and 40 mg/kg, respectively. Tongue lesions were made by acetic acid (bolus injection) on the ventral face. Ten mg/50 μL of l‐glutamine were applied at the site of the lesion in the treated group (*n* = 15) twice a day for 5 days; the control group (*n* = 15) received filtered water. On day 6, the animals were euthanized, the tongues removed, and processed for inflammatory and fibrogenic markers.

**Results:**

The treatment decreased myeloperoxidase (MPO) and *N*‐acetyl‐*β*‐glucosaminidase (NAG) activities, tumor necrosis factor alpha levels (TNF‐α), and mast cell numbers at the site of the lesions. The amino acid was able to decrease the number of Ki‐67 positive cells in the treated group. There was increased mucosal remodeling through collagen deposition in the treated group.

**Conclusion:**

Topical application of l‐glutamine acted on multiple axes of OM, modulating inflammation and tissue remodeling in immunosuppressed mice.

## 1 Introduction

Antineoplastic treatments cause oral mucositis (OM) in 40%–90% of the patients. This inflammatory condition often leads to severe pain, dysphagia, weight loss, malnutrition with consequent deterioration in the patient's quality of life. The severity of these lesions impairs continuity of treatment, which, in turn, will negatively affect cancer prognosis [1, 2, 3]. Lesions are characterized by white/yellow patches, erythema, bleeding, edema and ulcerations [3, 4, 5]. Several inflammatory biomarkers (cytokines, mediators and enzymes) have been shown to contribute to the severity of the lesions [6, 7]. Indeed, multiple systemic and topical treatments including physical modalities, laser therapy, pharmacological agents, natural products and nutritional supplementation have been used in the management of OM [2, 3, 8]. Of these, l‐glutamine stands out among the therapeutic approaches to treat/prevent OM complications being administered systemically or topically in different dosages, formulations and protocols [3, 9, 10, 11]. l‐glutamine is a cellular substrate and a source of energy not only for macrophages, lymphocytes and other cells of the immune system but also for rapidly proliferating stromal cells such as enterocytes and fibroblasts [12, 13]. These cells use l‐glutamine in a similar way to glucose, and it is also a precursor of intermediates in the Krebs cycle through glutamate and α‐ketoglutarate being directly involved in the process of immunological cell division, acid–base balance, in the transport of ammonia between tissues, and donation of carbon skeletons for gluconeogenesis [12, 13]. Reduced bio‐availability of l‐glutamine appears to impair several physiological functions, including immune response, antioxidant defenses and the regulation of inflammation induced by physical exercise or disease. The improvement of tissue repair processes in wounds of patients with increased protein catabolism, such as in seriously ill, burned, cancer or critical patients was observed after oral supplementation with l‐glutamine. This supplementation, recommended in these circumstances, appears to improve immune function, maintain intestinal integrity and nitrogen balance [13, 14, 15, 16, 17]. Moreover, the beneficial effects of l‐glutamine in pathological conditions have been, in part, attributed to glutathione (a subproduct of glutamine metabolism). Glutathione has been shown to suppress prostaglandin E2 (PGE2) synthesis, a potent inflammatory mediator [18]. In OM, administration of l‐glutamine has been shown to improve mucosal healing and to ameliorate mucosal immune function by impairing microbial invasion [19, 20, 21]. The protocol in which l‐glutamine was delivered as a swish and swallow liquid formulation was effective in reducing the frequency and duration of grade 3 and 4 in radiation‐induced OM [9, 10, 11]. Oral l‐glutamine was shown to slow down the beginning of OM and attenuate the severity of the injuries in patients of head and neck cancer receiving radio, chemotherapy or both [19, 20, 21, 22]. The assessment of l‐glutamine efficacy on OM is commonly based on standardized mucositis grading systems developed by international health organizations. The National Cancer Institute Common Terminology Criteria for Adverse Events, WHO and The Multinational Association of Supportive Care in Cancer and International Society of Oral Oncology (MASCC/ISOO) have published systematic reviews of the literature on various interventions for OM and evidence‐based clinical practice guidelines. Clinical and histopathological signs, including such as inflammation, edema, erythema, pain and ulceration are considered and graded most of them in humans [3, 4, 5].

While the above provide compelling evidence for the beneficial effects of this amino acid on these lesions in humans, there is a lack of information regarding its actions on the inflammatory and mucosa healing events underlying OM. Experimental animal models of diseases are useful tools to investigate mechanisms of drug actions, advance research and provide evidence for translational medicine. Using a model of 5‐fluorouracil‐induced OM in hamsters, it was shown that l‐glutamine and its derivative alanyl‐glutamine decreased two indices of inflammation (myeloperoxidase activity and glutathione stores) and improved mucosal healing [23], thereby providing initial evidence of the mechanism of action of this amino acid in OM in this animal model. To further establish mechanisms of action of l‐glutamine on OM, our objective was to examine the effects of topical application of l‐glutamine on a range of inflammatory components (enzyme activities, cytokines and mast cells) and remodeling components (Ki67, TGF‐β levels and collagen deposition) in acetic acid‐induced tongue lesions (OM‐like injury) in mice treated with 5‐FU. This chemotherapeutic agent, extensively employed as a pharmacological model, was used due to its immunosuppressive and catabolic actions that directly determine OM processes [24, 25]. We report here that topical application of l‐glutamine decreased inflammatory parameters and improved oral mucosal remodeling in immunosuppressed mice.

## 2 Materials and Methods

### 2.1 Animals

In this study, a total of 30 female C57BL/6 mice (7–8 weeks old weighing 17‐21g) were obtained from the Animal House (Bioterio Central) of the Universidade Federal de Minas Gerais (UFMG‐Brazil). The mice were housed individually, with free access to standard chow and water, and maintained at a 12:12 h light/dark cycle (lights on at 7:00 a.m.). To ensure ethical treatment, all procedures were designed to minimize animal distress and were performed in compliance with the UFMG Institutional Animal Committee guidelines (CEUA no. 204/2022).

### 2.2 Administration of Chemotherapeutic Drug (5‐FU)

Animals were prepared to receive intraperitoneal injections of the chemotherapeutic drug 5‐FU treatment for 3 non‐consecutive days. This protocol has been shown to induce leukopenia as previously reported [24, 25]. The administration started with an intraperitoneal application of 5‐FU at a dose of 60 mg/kg on the first day, 40 mg/kg on the third day, and 40 mg/kg on the fifth day. These days, in the experiment timeline, were denoted as −5, −3, and −1, respectively. There was no application of the chemotherapeutic agent on the second and fourth days. Before administration of 5‐FU, the animals were weighed and blood samples collected for quantitative determination of leukocytes. Body weight follow‐up was carried out on days 1, 3, and 6. Blood samples (20 μL) were collected by puncturing the tip of the tail in anesthetized animals (intraperitoneal injection of ketamine 80 mg/kg and xylazine 10 mg/kg). A total of 20 μL of blood was diluted in 400 μL of Türk's solution, and leukocyte counts (20 μL^3^) were performed using a Neubauer chamber, expressed as cells per microliter of blood. Leukocyte counts (*n* = 8) were obtained from a subset of animals selected from both experimental groups to confirm 5‐fluorouracil–induced leukopenia. This analysis was performed as a model validation step and not as a primary outcome; therefore, blood collection was limited to a subset of animals to minimize procedural stress.

### 2.3 Chemical Induction of Tongue Lesion

Twenty‐four hours after the last dose of 5‐FU, animals were anesthetized (intraperitoneal injection of ketamine 80 mg/kg and xylazine 10 mg/kg), and the ventral side of tongues exposed. A bolus injection of 5 μL of acetic acid 5% was administered in the ventral side of the tongue 2 mm below the mucosal surface using a microsyringe 31G‐0.5 mL. This protocol was based on the work by Shimamura et al. [26]. Having completed both protocols in all animals (5‐FU treatment and acetic acid tongue injection), animals were randomly allocated into two groups: 15 in the control and 15 in the glutamine‐treated group. Animals were monitored and the wounded areas examined.

### 2.4 Topical Application of l‐Glutamine Suspension and Removal of Tongue Biopsies

l‐Glutamine suspension (10 mg/50 μL) was applied using a pipette at the site of the lesion in the treated group twice a day for 5 full days; the control group received filtered water. The treatment started 24 h after the last dose of 5‐FU. Animals were immobilized for 30 min after the application to ensure that l‐glutamine was in direct contact with the lesion. The research team was trained in safe handling and restraint techniques to minimize stress. To reduce stress, animals were restrained only for the necessary period and closely monitored for signs of discomfort. Measures were taken to ensure their comfort and welfare, and after the application, animals were returned to their home cages and observed until fully relaxed. For the procedure, animals were held by the base of the tail; the loose skin at the nape of the neck was grasped to secure the head. The mouse was lifted into an upright position, and the mouth slightly open to expose the tongue for topic l‐glutamine administration. Care was taken to ensure normal breathing, pink coloration of the nose and feet for 30 min.

Treatment started 24 h after the acetic‐acid injection in the tongue. The dose of the compound was chosen based on the range used in experimental studies in animals [27, 28]. The procedure was carried out using Eppendorf tips. l‐glutamine was pre‐weighed and separated into labeled tubes, and it was diluted in filtered water at the time of application. Animals were humanely killed with an overdose of the anesthetic (10 x the anesthetic dose) after 5 days of l‐glutamine treatment (Figure S1). While still deeply sedated, to avoid pain and distress, tongues were removed from the animals. Biopsies of the tongue lesions were removed and processed for histopathological analysis, inflammatory and remodeling parameters. Seven tongue biopsies from each group were used for biochemical analyses and 5 for histological assessments. Before euthanasia, another blood sample following the same protocol was collected to confirm leukopenia. Death was confirmed by the absence of respiratory movements and cardiac activity prior to tissue collection.

### 2.5 Measurement of the Tongue Lesion Area

The tongue lesion area was assessed using excisional biopsies. Specimens were anatomically oriented and segmented. A macroscopic evaluation of the lesion area was first performed. Subsequently, the rostral portion of the tongue (anterior segment adjacent to the incisors), specifically the central (medial) region, was dissected longitudinally along the median plane. This procedure divided the ulcer into two symmetrical halves, which were then embedded together in the same paraffin block.

Serial histological sections of 5 μm thickness were obtained from standardized anatomical levels of the rostral portion of the tongue. Lesion boundaries were defined based on epithelial discontinuity, loss of normal tissue architecture, inflammatory cell infiltration, and damage to the underlying connective tissue. Lesion area was quantified using digital image analysis, and all measurements were performed by two evaluators blinded to the experimental groups.

Images were captured in slides (H&E staining) with a digital camera Q‐Color 5 connected to a microscopy Olympus BX43 equipped with a software Image‐Pro Plus 7.0 Media Cybernetics, USA. The resolution was set at 2560 × 1920 pixels (20× magnification).

### 2.6 Histological Analysis

Biopsies of tongue lesions excised from each group of mice (*n* = 5) were dissected and fixed in formalin (10% w/v in isotonic saline). Sections (5 μm) were stained with Hematoxylin and Eosin (H&E) for evaluation of the area of the tongue lesions and the mucositis parameters. Images were analyzed to identify inflammatory areas (cells with nuclei) from necrotic regions (cells without nuclei). Histological images were converted to optical density (Sum OD) to delimit the perimeter of the total lesion area (mm^2^) and the necrotic region in both groups. The injured areas were delimited by drawing a red line around the total lesion area and a yellow line around the necrotic area.

Features of mucositis were quantified in the entire sections of biopsies according to the extent of tissue alterations. Our grading system was modified from previous publications from animal models [23, 25]. Epithelial alterations were assessed separately: presence of proliferative alterations (hyperkeratosis and hyperplasia) was assigned 1 point; presence of ulceration was assigned 2 points; and presence of both ulceration and proliferative alterations was assigned 3 points. Inflammatory alterations: Inflammatory cells (0–3), edema (0–3), hemorrhage (0–3), were scored, and the final sum of each group was analyzed. The maximum score (7–12 points) was considered 100% (intense tissue alterations), score (3–6 points) between 50% and 25%, moderate alterations, and below 25% score (0–3 points) mild tissue alterations. After the evaluation, the scores assigned by the two evaluators were added and the average score was calculated for each animal. Ten fields per slide were used for data analysis at 10× magnification. Images were acquired with a Q‐Color 5 camera attached to an Olympus BX43 microscope and analyzed in QuPath (version 0.3.2).

Dominici staining was employed to quantify mast cell population, identified by features of metachromasia. Images of 20 fields from histological cross‐sections from each tongue were captured with a panchromatic objective lens (×40) in an optical microscope (final magnification 400×).

Picrosirius‐red staining was used followed by polarized light microscopy to identify and quantify collagen fibers. Images of sequential cross‐sections of each biopsy were obtained from 20 fields (area = 4795 μm^2^) and captured using a panchromatic objective lens (40×) on an Olympus BX43 light microscope (final magnification = 400×). Images were digitized and analyzed using Image Pro‐Plus 7.0 software with a final resolution of 2560 × 1920 pixels (Media Cybernetics Inc., USA).

### 2.7 Immunohistochemistry (IHC) for Ki‐67

IHC reaction for the detection of Ki67 was performed using anti‐human Ki‐67 (BD 550609). Tissue sections (5 μm) (*n* = 3) were dewaxed and a heat‐induced antigen retrieval was performed using a pressure cooker with citrate buffer (pH 6), then slides were cooled for 30 min in the same buffer. Sections were incubated for 15 min with Peroxidase Blocker from Novolink Polymer Detection Systems (Leica) and protein blocking was performed with solution from the same IHC kit. Slides were incubated overnight at 4°C with primary antibodies rabbit anti‐Ki‐67 (1:100) (Cell Signaling Technology, 12 202); followed by incubation with Post Primary and Polymer solutions. Peroxidase activity was visualized using DAB chromogen. The absence of staining in negative controls, where the primary antibody was omitted, confirmed specificity. Sections were then counterstained with hematoxylin. For morphometric analysis, images of stained cells from all cross‐sectional fields using a 100× planapochromatic objective (final magnification = 1000×) were counted in light microscopy. The images were digitized through an Olympus microcamera and transferred to an analyzer (QCapture).

### 2.8 Determination of Myeloperoxidase and *N*‐Acetyl‐*β*‐d‐glucosaminidase Activities

Neutrophil infiltration into the tongue lesions was assessed by analyzing myeloperoxidase (MPO) activity, a lysosomal hemoprotein found in the azurophilic granules in neutrophils as previously described [29, 30, 31].

Excisional Tongue biopsies from 7 mice in each group were used for this assay. Pellets from centrifugation of tissue homogenates were divided into two portions, a part of the corresponding pellet was weighed, homogenized in pH 4.7 buffer (0.1‐M NaCl, 0.02‐M NaPO_4_, 0.015‐M NaEDTA), and centrifuged at 12 000*g* for 10 min. The pellets were then resuspended in 0.05‐M NaPO_4_ buffer (pH 5.4) containing 0.5% hexadecyltrimethylammonium bromide (HTAB) followed by three freeze thaw cycles using liquid nitrogen. MPO activity in the supernatant was assayed by measuring the change in absorbance (optical density; OD) at 450 nm using tetramethylbenzidine (1.6 mM) and H_2_O_2_ (0.3 mM). The reaction was terminated by adding 50 mL of H_2_SO_4_ (4 M). Results were expressed as a change in OD per gram of wet tissue. The remaining part of the pellet was used to quantify mononuclear cells accumulation in the biopsies by measuring the levels of the lysosomal enzyme *N*‐acetyl‐*β*‐d‐glucosaminidase (NAG) present in high levels in activated macrophages [29, 30, 31]. Briefly, pellets were weighed, homogenized in NaCl solution (0.9% w/v) containing 0.1% v/v Triton X‐100 (Promega, Madison, WI, USA), and centrifuged (3000*g*; 10 min at 4°C). Samples (100 μL) of the resulting supernatant were incubated for 10 min with 100 μL of *p*‐nitrophenyl‐*N*‐acetyl‐*β*‐d‐glucosaminide (Sigma‐Aldrich, St. Louis, MO, USA) prepared in citrate/phosphate buffer (0.1‐M citric acid, 0.1‐M Na_2_HPO_4_; pH 4.5) to yield a final concentration of 2.24 mmol. The reaction was stopped by the addition of 100 μL of 0.2‐M glycine buffer (pH 10.6). Hydrolysis of the substrate was determined by measuring the absorption at 400 nm. Results are expressed as nmol per gram of wet tissue.

### 2.9 Measurement of TNF‐α, and TGF‐β Production

Supernatants of the tongue biopsies used for inflammatory enzyme determination (*n* = 7) were employed for cytokines evaluation. They were homogenized in PBS pH 7.4 containing 0.05% Tween and centrifuged at 10 000*g* for 30 min. The levels of the cytokines in the supernatant from each tongue (50 μL) were measured using Immunoassay Kits (R and D Systems, USA) and following the manufacturer's protocol to each cytokine. Briefly, dilutions of cell‐free supernatants were added in duplicate to ELISA plates coated with a specific murine monoclonal antibody against cytokine, followed by the addition of a second horseradish peroxidase‐conjugated polyclonal antibody, also against cytokine.

After washing to remove any unbound antibody‐enzyme reagent, a substrate solution (50 μL of a 1:1 solution of hydrogen peroxide and tetramethylbenzidine 10 mg/mL in DMSO) was added to the wells. Color development was halted after 20 min incubation with 2 M sulfuric acid (50 μL) and the intensity of the color was measured at 540 nm on a spectrophotometer (Thermoplate). Standards were 0.5‐log10 dilutions of recombinant murine cytokines from 7.5 to 1000 ρg/mL (100 μL). The threshold of sensitivity for each chemokine is 15.625 ρg/mL. The results were expressed as ρg cytokine per mg wet tissue.

### 2.10 Statistical Analysis

All data are presented as mean ± SEM for normally distributed variables or as median (interquartile range, IQR) for non‐normally distributed variables. The normality of the data was assessed using the Shapiro–Wilk test, and homogeneity of variances was evaluated with Levene's test. When the assumptions of normality and homoscedasticity were met, parametric tests were applied (Student's *t*‐test unpaired or paired for comparisons between two groups, one‐way or two‐way ANOVA followed by appropriate post hoc tests for multiple groups). Outliers were identified and excluded using the ROUT method (*Q* = 1%) through the “Identify Outliers” function in GraphPad Prism 8.2.1 (GraphPad Software, San Diego, CA, USA). The complete statistical outputs generated by GraphPad Prism, including test statistics and exact *p*‐values, are provided as Tables S1–S11. All experiments were performed in at least three independent biological replicates.

## 3 Results

### 3.1 Effects of 5‐FU on Leukocytes Number and Body Weight

**FIGURE 1:**
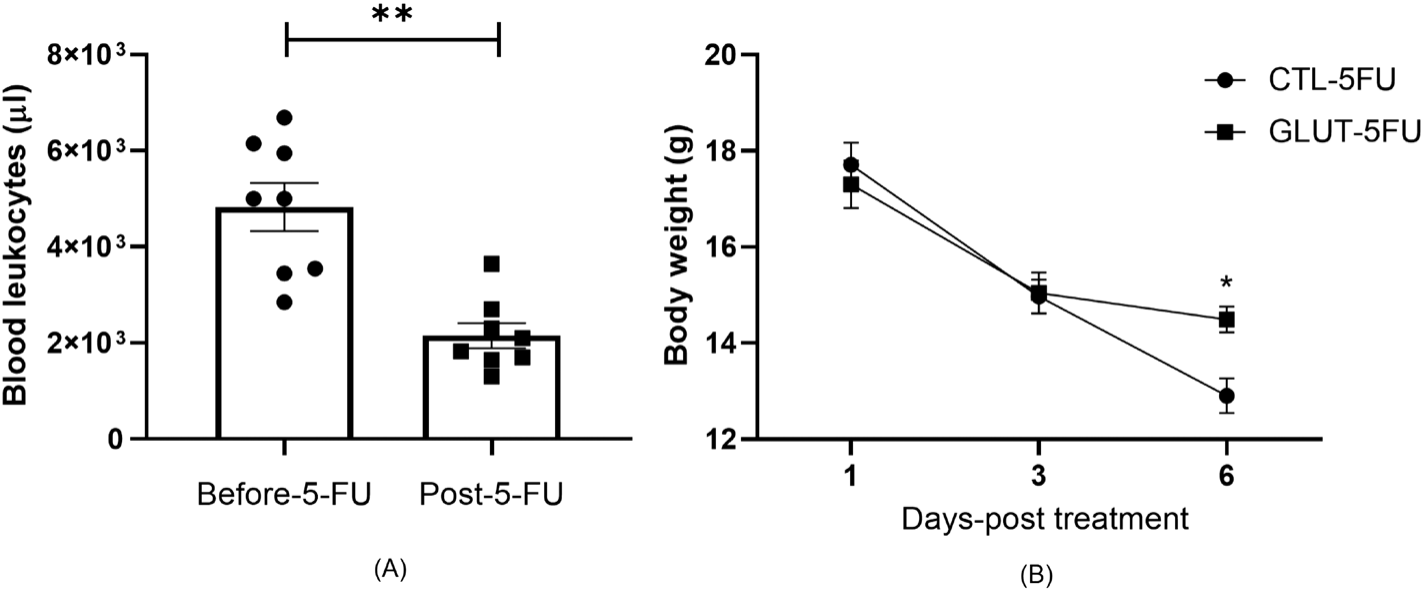
Effects of 5‐FU on blood leukocytes number and body weight. Administration of the chemotherapeutic agent caused a decrease in blood leukocytes/μL^3^ (55.4%) compared with the samples before 5‐FU administration (A; *n* = 8 in each group) Student's *t*‐test pared, *p* = 0.004. Animals in both groups lost weight. At day 6, the loss was more accentuated in the control group (B; *n* = 12–15 animals in each group) 2 way ANOVA *p* = 0.049. Values are means ± SEM.

The experimental model employed three non‐consecutive doses of 5‐fluorouracil (5‐FU) to induce immunosuppression, resulting in a reduction in leukocyte count in animals prior to treatment. The initial counting in blood samples, before 5‐FU, was 4830 ± 499 leukocytes/μL^3^ (*n* = 8). On day 6, after treatment, animals were euthanized; leukocyte number/μL^3^ was 2154 ± 262 (*n* = 8), which is a 55.4% reduction, *p* = 0.004 (Figure 1A). Animals in both groups (control *n* = 15 and l‐glutamine‐treated; *n* = 15) lost weight at the end of the experiments. At the beginning of the experiments, control animals weighed 17.71 ± 1.80 (*n* = 15); the treated group weighed 17.30 ± 1.85 (*n* = 15). The final weight of the control was 12.9 ± 1.15 (*n* = 10), and the treated group was 14.49 + 0.93 (*n* = 12). There was a significant difference (*p* = 0.049) between the groups in the final weight (Figure 1B).

### 3.2 Macro and Histomorphological Evaluation of the Tongue Lesions

**FIGURE 2:**
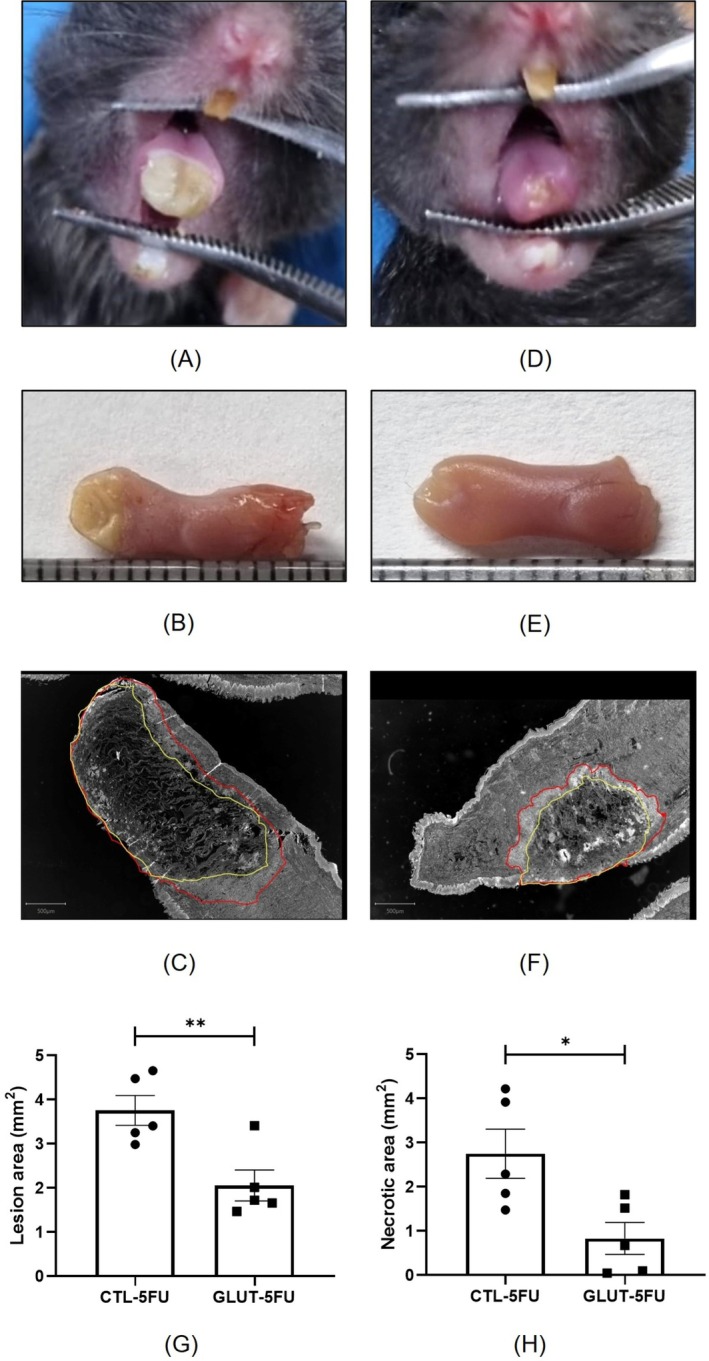
Representative images in situ and histology of the tongue lesions (Control A, B and C; Treated D, E and F). The tongue lesion in the control animal was apparently more severe than that in the l‐glutamine‐treated animal. The excised tongue from the control mice was thinner/atrophic/hypochromic compared with that of the treated animal. The injured areas were delimited by drawing lines around the perimeters (C, F). Total lesion (red line) and necrosis (yellow line) area (mm^2^) of each group (G, H). The mean measurements (mm^2^) in the control total lesion and necrotic area were greater compared to the treated group. Values are means ± SEM of five animals in each group. *p = 0.020, **p = 0.008, Student *t*‐test. Planapochromatic objective (4×) in light microscopy (final magnification = 40×). Scale bar, 500 μm.

In situ images of tongues from control and treated mice showed tissue damage and inflammation. Tongue lesions observed macroscopically in control animals were larger than those in animals treated with l‐glutamine. The excised tongue from the control mice was thinner/atrophic/hypochromic compared with that of the treated animal (Figure 2A–F).

The lesion and necrotic areas were larger in the control group. The values of the mean lesion area measurements (mm^2^) were 3.75 ± 0.33 control versus 2.05 ± 0.34 l‐glutamine‐treated (*p* = 0.008). The necrotic area (mm^2^) was 2.76 ± 0.55 in the control versus 0.83 ± 0.36 in the treated group (*p* = 0.020) (Figure 2G,H).

**FIGURE 3:**
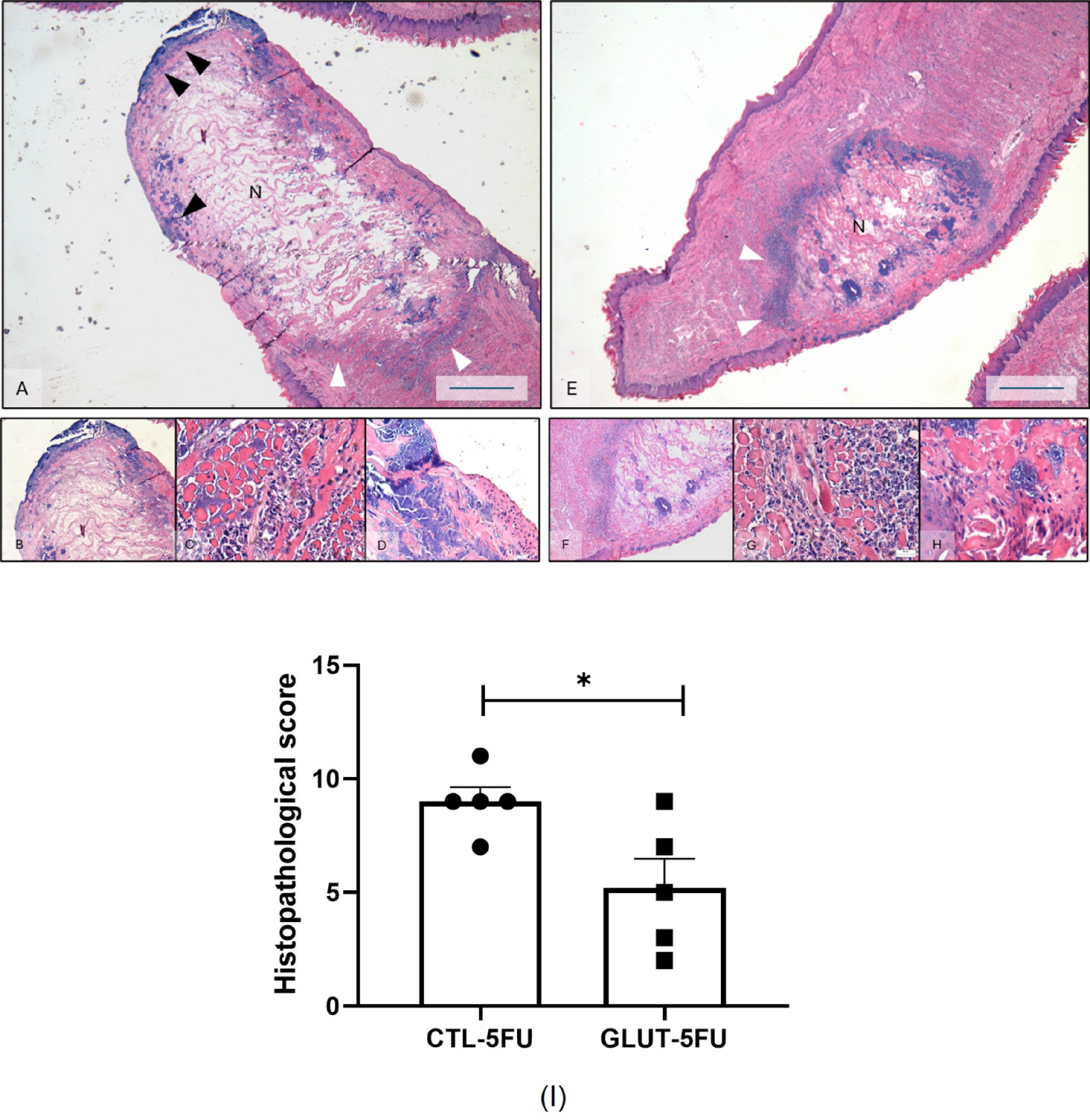
Representative histological sections of tongue biopsies stained with Hematoxylin and Eosin (H&E) and histopathological score. In tongue lesions, inflammatory cells, erythema, hemorrhage, necrotic area and ulceration are seen in both groups. In the image of the control mouse, these features were more pronounced (control A–D; treated E–H). These features were identified morphologically: (A) Overview of extensive tongue lesions in the 5‐FU + vehicle‐treated group. (B) Necrotic region at the tip of the tongue. (C) Area with inflammatory infiltrate predominantly consisting of polymorphonuclear cells and necrosis of muscle fibers. (D) Presence of parakeratosis. (E) Overview of the tongue in the 5‐FU + glutamine‐treated group, showing reduced affected area. (F) Ulceration, polymorphonuclear inflammation, and mild parakeratosis. (G) Delimitation of the inflammatory infiltrate. (H) Area of parakeratosis. (N) indicates a necrotic region; Black arrowhead indicates ulceration; white arrowhead indicates a circumscribed region of inflammatory cells. The histopathological score of the inflammatory parameters analyzed was less in the treated group than in the control (I). The data were considered nonparametric, and statistical analysis was performed using the Student *t*‐test, with p* = 0.028. Values are medians ± SEM of five animals in each group. Planapochromatic objective (40×) in light microscopy (final magnification = 400×). Scale bar, 50 μm.

Histomorphological analysis (H&E staining) was employed to evaluate the microscopic features of the mucositis in control and treated groups. It was observed that in the area of the lesions in both groups there were inflammatory cells, blood vessels, hemorrhage, edema, extracellular matrix fibers, epithelial ulcerations and an eosinophilic transudate. However, lesion size was significantly reduced as a result of drug intervention compared with untreated controls; in contrast, these features were more pronounced in the control group (Figure 3A–D) compared to the treated group (Figure 3E–H). The histopathological score of mucositis severity analyzed was lower in the treated group 5.20 ± 1.28 versus that in the control 9.0 ± 0.63 (*p* = 0.028) (Figure 3l).

### 3.3 Soluble Inflammatory Markers Evaluation and Mast Cell Number

**FIGURE 4:**
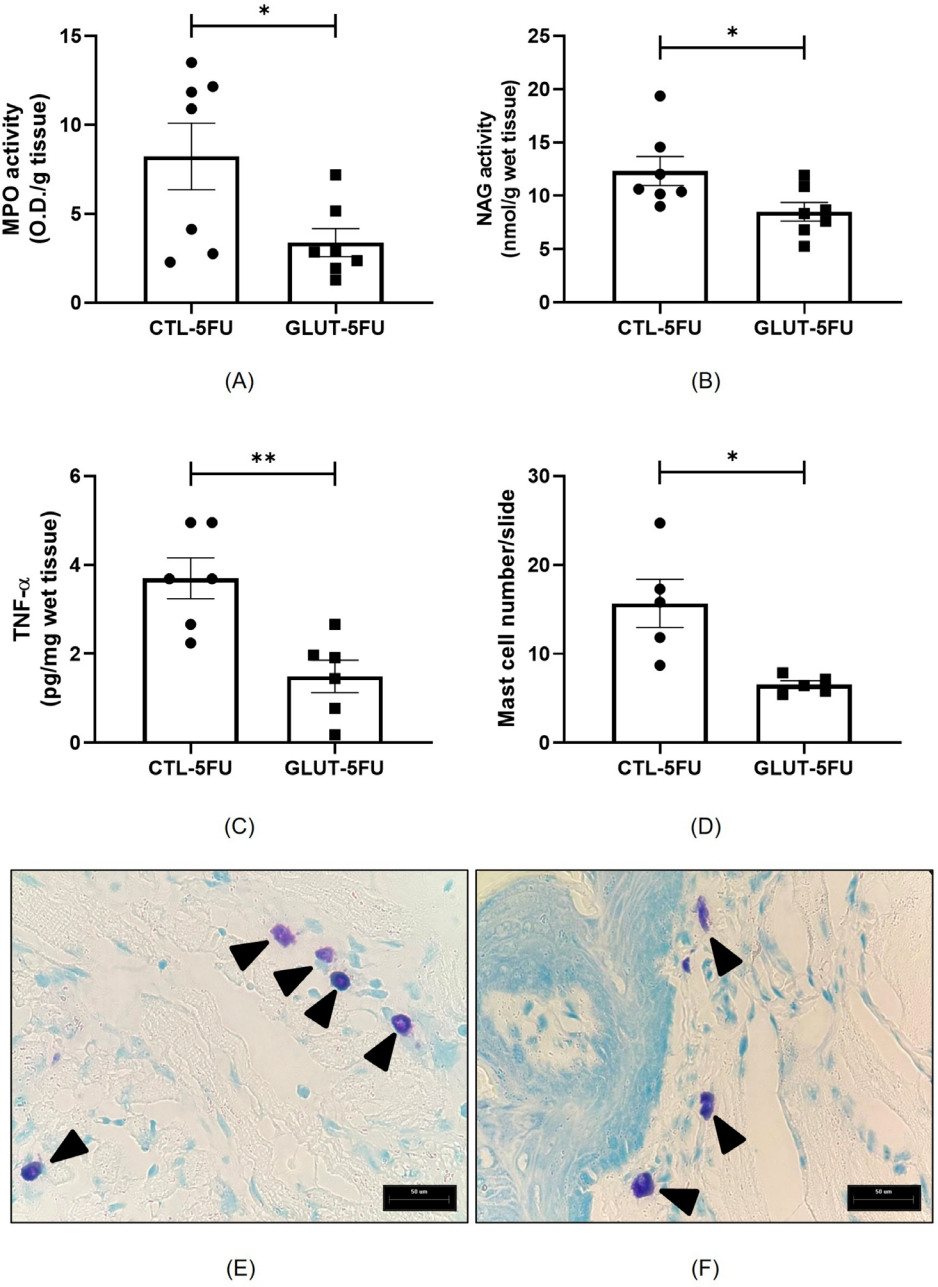
Effects of l‐glutamine on inflammatory markers and mast cell number. The production of all inflammatory markers myeloperoxidase‐MPO (A) *p* = *0.0337; *N*‐acetyl‐*β*‐d‐glucosaminidase‐ NAG (B) *p = 0.0357; tumor necrosis factor alpha‐ TNF‐α (C); were lower in the treated group (*n* = 7) **p = 0.0037. The mast cells counting, arrowheads (black arrows) stained with Dominici staining was lower in the treated group (*n* = 5) *p = 0.0106 (D–F). Planapochromatic objective (40×) in light microscopy (final magnification = 400×). Scale bar, 50 μm. Values are means ± SEM, Student *t*‐test.

Inflammation of the tongue was determined by evaluating MPO, NAG activities and TNF‐α levels (Figure 4A–C). In addition, the number of mast cells was quantified in the control and treated groups (Figure 4D–F). The values for MPO were 8.22 ± 1.86 in control versus 3.38 ± 0.78 (O.D/g wet tissue) *p* = 0.034 in the treated group and NAG values were 12.31 ± 1.35 control versus 8.50 ± 0.86 (nmol/g wet tissue) *p* = 0.036 in the l‐glutamine group. The treatment decreased TNF‐α production (pg/mg tissue) in the treated group (control 3.5 ± 0.38 versus 1.46 ± 0.31), *p* = 0.004 *n* = 7. The number of mast cells/slide was also less in the treated group, 6.54 ± 0.44 versus that in the control 15.67 ± 2.71, (*n* = 5), *p* = 0.011.

### 3.4 Remodeling Markers Evaluation

**FIGURE 5:**
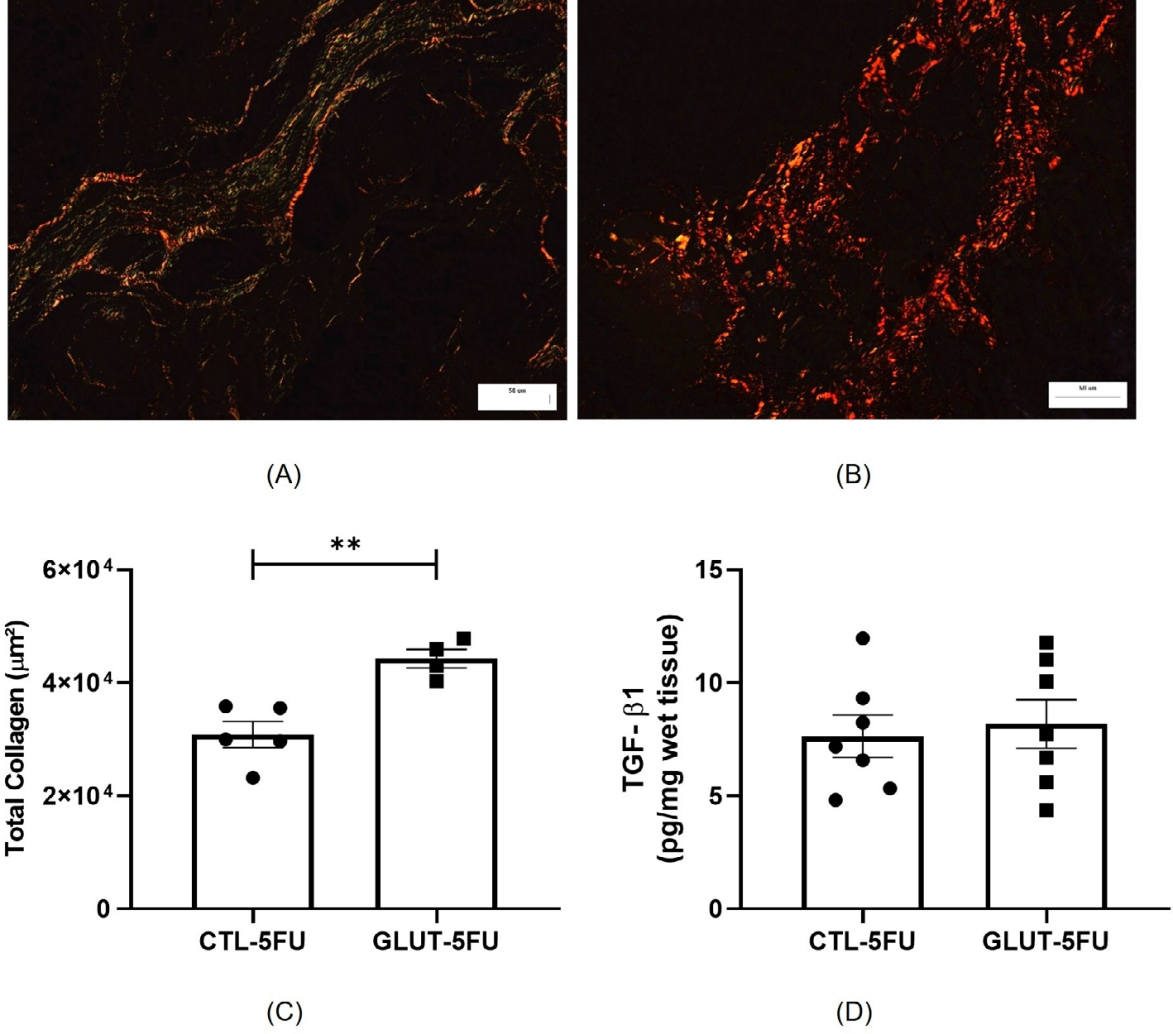
Effects of l‐glutamine on remodeling parameters. Representative photomicrographs of collagen fibers visualized by Picrosirius red staining showing the control (A) and the l‐glutamine‐treated (B). The total collagen area was larger in the treated group compared with the control group **p = 0.0029 (*n* = 5). TGF‐β1 levels were similar in both groups (D) *p* = 0.7084 (*n* = 7 animals). Results are expressed as mean ± SEM. Student's *t*‐test. Planapochromatic objective (40×) in light microscopy (final magnification = 400×). Scale bar = 50 μm.

**FIGURE 6:**
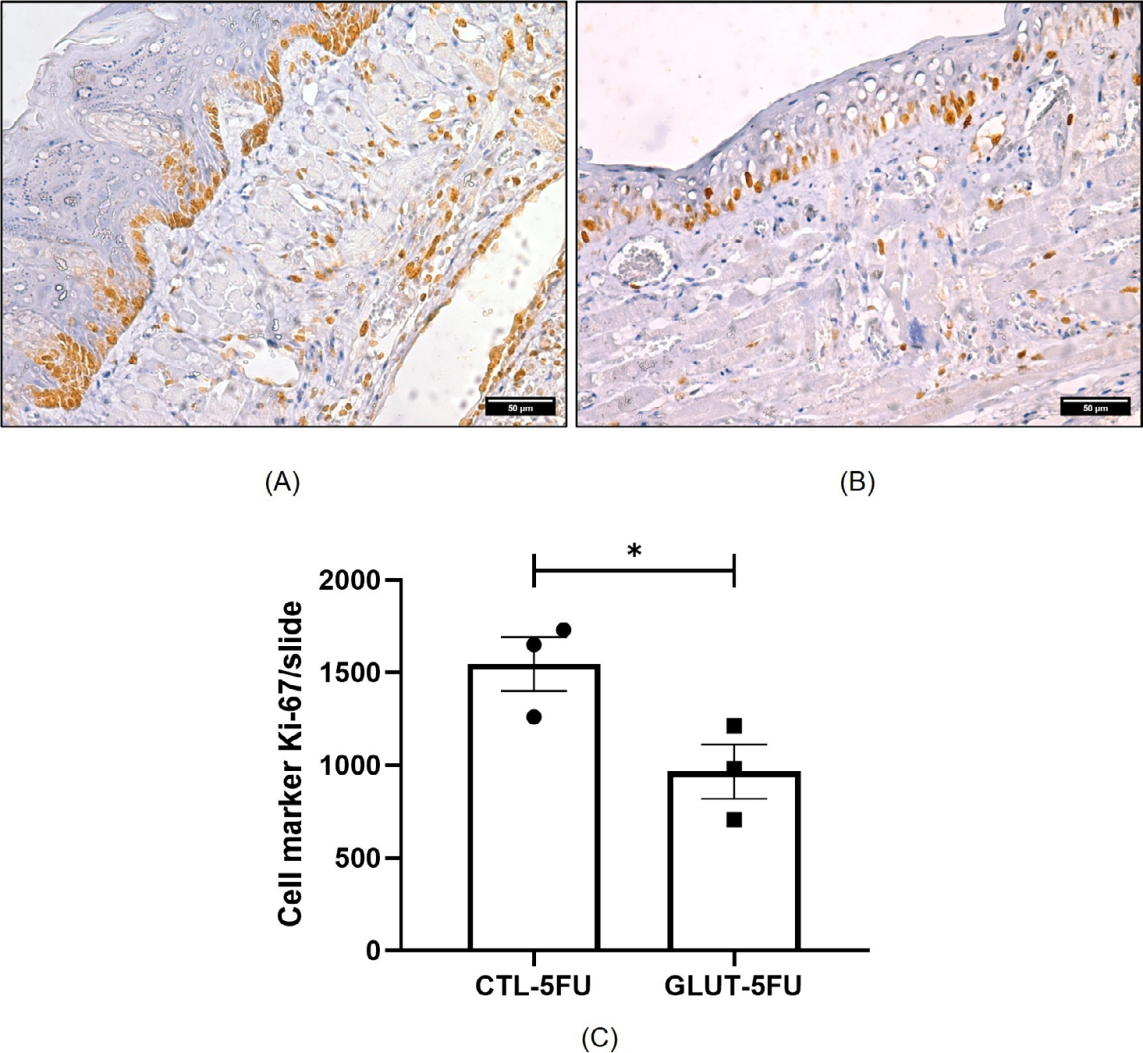
Effect of l‐glutamine on the proliferative marker. Representative micrographs of tongue lesions (A, control; B, treated) immunostained with Ki‐67. More positive cells were observed in the control implant compared with the treated (C). Values are means ± SEM; *p = 0.0479; Student's *t*‐test, *n* = 3. Planapochromatic objective (40×) in light microscopy (final magnification = 400×). Scale bar, 50 μm.

Topical application of l‐glutamine on the tongue lesions increased collagen synthesis/deposition (Picrosirius red staining). Interestingly, collagen type I (reddish color) was predominant in the treated group, whereas more immature collagen (greenish color) was predominant in the control group (Figure 5A–D). The total collagen area in the treated group was 44 277 ± 1654 versus 30 866 ± 2315 μm^2^ in the control group, *p* = 0.003 (*n* = 5). Levels of the pro‐fibrogenic cytokine TGF‐β were not different between both groups, control 7.63 ± 0.93 pg/mg versus 8.18 ± 1.07 pg/mg in the treated group *p* = 0.708 (*n* = 7). We performed Ki‐67 immunostaining to determine whether the amount of cell proliferation in the control group (Figure 6A–C) differed from the treated group. The number of Ki‐67 positive cells was 1567 ± 145 (control) versus 966 ± 146 (treated) *n* = 3, *p* = 0.048.

## 4 Discussion

OM, as a side effect of cancer therapy, is a severe health problem that occurs in about 40%–90% of patients with consequent deterioration in their quality of life [1, 2, 3, 4, 5]. It is characterized by inflammation and ulceration of the oral mucosa inflicting pain, dysphagia, weight loss, malnutrition as a result of the loss of regenerative ability of the oral epithelial cells. A wide range of therapeutic approaches used have provided clinical evidence of the efficacy of some products ameliorating the signs and symptoms of OM [3, 4, 10]. Glutamine, for instance, previously reported for improving tissue repair in seriously ill, burned, cancer or critical patients, has been used in the management of OM [13, 14, 15, 16]. The assessment of l‐glutamine efficacy on OM in humans and experimental animals is based on clinical and histopathological mucositis grading systems described initially by Sonis et al. [24, 25] and modified by a number of Health Organizations/Researchers [3]. We reasoned that evaluation of cellular and molecular biomarkers would identify mechanisms and effectors involved in the actions of l‐glutamine on OM. Using topical application of l‐glutamine in acetic acid‐induced tongue lesions in mice treated with 5‐FU, we examined a range of inflammatory and remodeling parameters. In our Institution, several mice strains (inbred and outbred) are readily available. They are small, less expensive, easy to handle and to maintain. In addition, they have proved to be a suitable preclinical model of chemotherapy‐induced alimentary tract mucositis [32]. We chose to use female mice because in humans female patients have a greater risk of developing severe mucositis when treated with 5‐FU [33]. The experimental animal model of OM differs from the clinically observed oral mucositis in humans. While this condition is a common severe side effect of 5‐FU treatment in humans characterized by white/yellow patches, inflammation, erythema, bleeding, edema and ulceration in the oral cavity [3, 4, 5], in mice the same chemotherapeutic agent does not cause ulcers in the alimentary cavity [26]. To overcome this limitation and to optimize a suitable pre‐clinical model of chemotherapy‐induced oral mucositis, a model based on the combination of systemic immune suppression and mucosal chemical (acetic acid) insult was developed in mice and proved effective in mimicking oral OM in humans [26]. It is also relevant to mention that the tongue lesions caused by acid injection differ intrinsically from the mucosal lesions in cancer patients treated with the chemotherapeutic agent. However, the two types of lesions share common pathological features (edema, inflammation, ulceration). Despite the histopathological similarities between experimental chemotherapy‐induced mucositis in animals and in humans, it is possible that the distinct types of injuries may have influenced lesion development, inflammatory markers, and healing in the damaged tissue.

In the first set of results, we showed that 5‐FU treatment reduced blood leukocyte counting, confirming the immunosuppressant effects of this chemotherapeutic agent [24, 25]. The metabolic data, body weight loss, was observed in both groups. It is reported as a side effect of 5‐FU [32, 34]. Interesting, weight loss in the treated group was less than in the control group at the end of the experiment. This difference may be related to the severity of lesions, as observed using our scoring system. It is possible that topical application of l‐glutamine exerted a protective effect on the lesions allowing more food ingestion/deglutition. Protective effects of glutamine in OM have been reported in humans [9, 10, 11].

In situ analysis of the tongue lesions and measurements of the damaged areas on day 6 post‐injury (5 days of topical l‐glutamine application) showed that the size of the damaged areas was smaller in the treated group. Other macroscopic features such as erythema, hyperaemia, hemorrhagic areas, and epithelial ulcerations were seen, but less pronounced in the treated group. The presence of these signs has been proposed as functional criteria in OM in humans [3, 35]. In the histopathological analysis of the lesions (H&E staining), it was possible to identify inflammatory cells, erythema, ulceration, hemorrhagic and necrotic areas, and hyperkeratosis. These features were less abundant in the treated group compared with that of control according to our score system, which was based on the grading systems by Sonis et al. [25] and by Leitao et al. [23]. Our finding is in agreement with the work by Leitao showing that oral Glutamine and alanyl‐glutamine reduced inflammatory parameters in oral mucositis in hamsters according to their grading system. Therefore, our findings have not only identified and graded OM hallmarks but also shown the modulatory effects of topical application of l‐glutamine on tongue lesions in mice. Several studies have demonstrated the beneficial effects of glutamine in OM in human and animal models [3, 20, 21, 22, 23, 35]. We then analyzed 4 inflammatory markers (MPO and NAG activities, TNF‐α production, and mast cell number) in the tongue lesions. All the markers analysed were reduced in the treated group compared with the control group. The biomarkers MPO and TNF‐α have been identified in OM both in human and experimental animals [3, 36]. However, we found only one study reporting that oral glutamine decreased MPO activity in oral mucositis in hamsters [23]. While the anti‐inflammatory effects of glutamine are well established in various pathological conditions, its effects on OM are less well understood [28, 37, 38, 39]. It is possible that l‐glutamine may have downregulated the activity/recruitment of the inflammatory cells present in the mucositis microenvironment, thus reducing/protecting the damaged tissue. We have also examined the number of mast cells in the tongue lesions. Mast cells are rich sources of various cytokines, including TNF‐α and inflammatory mediators, and have been studied in radiation‐induced oral mucositis in hamsters [25, 40]. It is likely that their activation at the site of the injury may have amplified the severity of the mucositis in the untreated group. It was interesting to observe that l‐glutamine was able to decrease the number of this cell type in OM in mice. We found no study that has reported the effect of this amino acid on mast cells recruitment/activation on OM in mice. Our findings on the effects of topical application of l‐glutamine on inflammatory events of OM in mice disclose its action on multiple targets of the process.

In our study, we evaluated different remodeling markers in the tongue biopsies of control and treated mice with l‐glutamine (collagen deposition‐ types 1 and 3, TGF‐beta levels, Ki67 proliferative marker). Total collagen area (Picrosirius red staining) was bigger in the l‐glutamine group. More mature collagen (type I, reddish color) was observed in the lesions of treated animals compared with that of control mice (type III, immature collagen). Leitao et al. [23] also reported that glutamine accelerated mucosal healing by evaluating histological parameters in H&E staining sections. In another study, oral administration of l‐glutamine attenuated cutaneous wound healing in rats by increasing collagen synthesis [41]. It is possible that l‐glutamine may have accelerated collagen maturation/early fibrosis in the tongue lesions. Given that we analysed one time point (day six post‐injury) in the OM process, our finding may indicate modulation of extracellular remodeling rather than definitive evidence of improved healing.

We evaluated the levels of TGF‐β, a cytokine known to modulate collagen synthesis/deposition which is directly involved in physiological and pathological wound healing [42]. Its levels were not different between l‐glutamine treated and control group. It is likely that l‐glutamine may have acted through its metabolites rather than through the fibrogenic cytokine. It has been documented that products of l‐glutamine (proline, hydroxyproline, glycine, alpha‐ketoglutarate, glutamate) are essential for collagen synthesis in various wounded tissues [43, 44, 45]. Whether the increased collagen deposition has occurred through (glutamine metabolites) in experimental chemotherapy‐induced mucositis in mice remains to be investigated. The proliferative activity of tongue biopsies was less intense in the treated group at day 6 post‐treatment (lower Ki67 positive cells), suggesting that l‐glutamine may have reduced the severity of the tongue lesions and/or reduced the inflammatory process. This and other markers have been extensively used to assess proliferative activity in OM cells [46, 47].

To our knowledge, there are no reports on the effects of topical application of l‐glutamine on collagen deposition in OM. Thus, we show for the first time a modulatory effect of l‐glutamine on remodeling of oral lesions. This finding may be clinically relevant in the management of OM.

One limitation of this study was the fact that the animals treated with 5‐FU had no cancer. Therefore, it remains to be investigated whether the beneficial effects of topical l‐glutamine would be observed on OM in animals bearing cancer under 5‐FU treatment. It would also be relevant to evaluate the baseline levels of the tongue tissue features (collagen organization, basal levels of inflammatory and fibrogenic cytokines, proliferative activity, Ki‐67 expression) in immune competent mice to establish the influence of chemotherapeutic agents in physiological parameters. It is clear that the missing healthy groups limited the mechanistic interpretation and translational relevance of our study. Certainly, future experiments will be carried out to examine these issues. Regardless of these limitations, our study confirms and extends the beneficial effects of l‐glutamine on OM and indicates that they are associated with down‐regulation of inflammatory targets and up‐regulation of collagen synthesis.

## Author Contributions

**Juliana Francisca Grossi Heleno**, **Paula Peixoto Campos**, and **Silvia Passos Andrade:** conceptualization. **Juliana Francisca Grossi Heleno**, **Igor de Campos Fontes**, **Leticia Cristine Cardoso dos Santos**, **Victor Prokop Gonçalves Campos**, **Iury Berckmann Freitas Gomes:** methodology. **Juliana Francisca Grossi Heleno**, **Igor de Campos Fontes**, **Leticia Cristine Cardoso dos Santos**, **Paula Peixoto Campos**, and **Silvia Passos Andrade:** validation. **Igor de Campos Fontes**, **Leticia Cristine Cardoso dos Santos**, and **Paula Peixoto Campos:** formal analysis. **Juliana Francisca Grossi Heleno**, **Igor de Campos Fontes**, **Leticia Cristine Cardoso dos Santos**, and **Paula Peixoto Campos:** investigation. **Paula Peixoto Campos:** resources. **Paula Peixoto Campos** and **Silvia Passos Andrade:** data curation. **Paula Peixoto Campos**, **Derek William Gilroy**, and **Silvia Passos Andrade:** writing – original draft preparation. **Paula Peixoto Campos**, **Silvia Passos Andrade**, **Derek William Gilroy**, and **Leticia Cristine Cardoso dos Santos:** writing – review and editing. **Paula Peixoto Campos**, **Silvia Passos Andrade**, **Derek William Gilroy** and **Leticia Cristine Cardoso dos Santos:** visualization. **Paula Peixoto Campos** and **Silvia Passos Andrade:** supervision. **Paula Peixoto Campos** and **Silvia Passos Andrade:** project administration. **Paula Peixoto Campos:** funding acquisition. All authors have read and agreed to the published version of the manuscript.

## Funding

This work was supported by the Minas Gerais Research Support Foundation (FAPEMIG RED‐00570‐16, RED‐00213‐23) and Benjamin Guimarães Foundation, Teaching and Research Center at Hospital of Baleia, Minas Gerais, Brazil.

## Disclosure

The animal study protocol was approved by the Biological Institute of Biological Science Institutional Animal Welfare Committee (process number: CEUA n° 204/2022).

## Conflicts of Interest

The authors declare no conflicts of interest.

## Supporting information


**Figure S1:** Experimental design of the oral mucositis model.

**Table S1:** Effects of 5‐FU in blood Leukocytes number.
**Table S2:** Effects of 5‐FU in body weight.
**Table S3:** Lesion area.
**Table S4:** Necrotic area.
**Table S5:**. Histopathological score (HE).
**Table S6:** Effects of l‐Glutamine on NAG activity.
**Table S7:** Effects of L‐Glutamine on TNF‐α production.
**Table S8:** Effects of L‐Glutamine on the mast cell number.
**Table S9:** Effects of L‐Glutamine on collagen fiber quantification.
**Table S10:** Effects of L‐Glutamine on TGF‐β1 levels.
**Table S11:** Effect of L‐Glutamine on the proliferative marker ki67.

## Data Availability

The data that supports the findings of this study are available in the Supporting Information of this article.
