# Supplementary figures and images for: Dual Beneficial Effects of Topical l‐Glutamine on Oral Mucositis in 5‐Fluorouracil‐Treated Mice
Source: J Oral Pathol Med. 2026 Apr 7;55(6):705–17. doi: 10.1111/jop.70135 (PMC13333535; doi:10.1111/jop.70135)

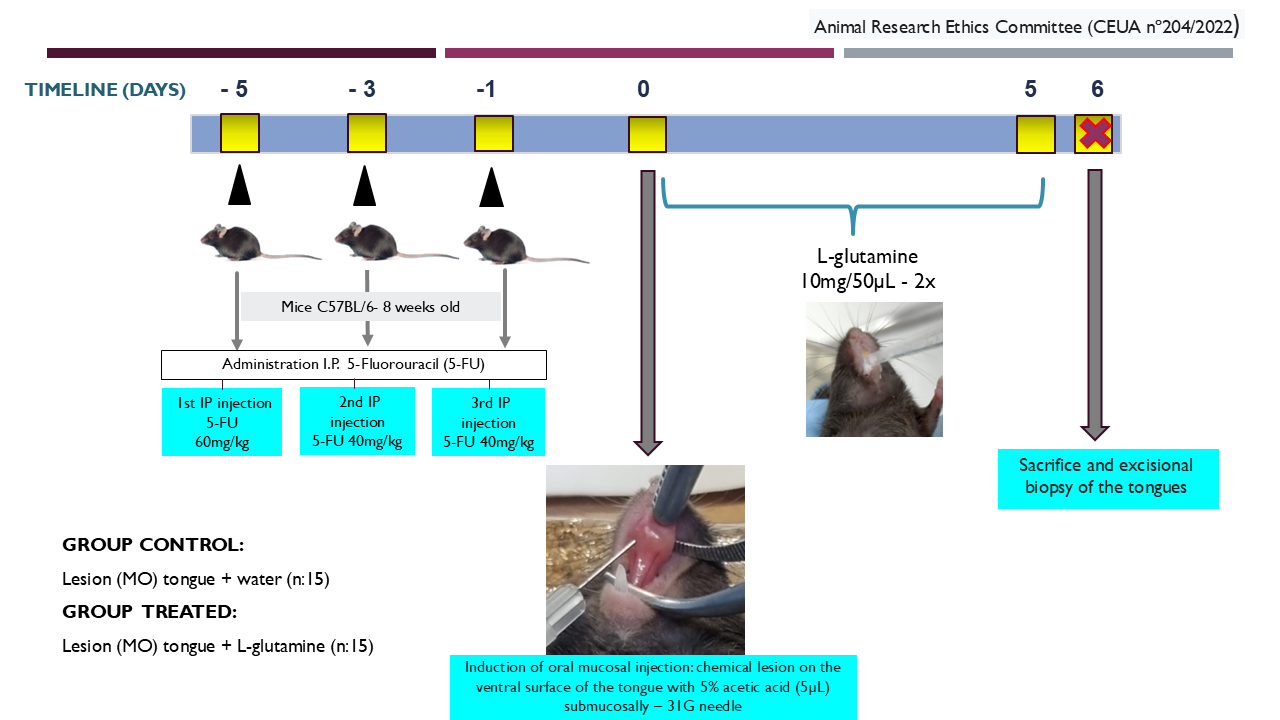

Supplement: Supplementary file 1 — Figure S1: Experimental design of the oral mucositis model. [file JOP-55-705-s002.tif]
