# Supplementary material for: Dual Beneficial Effects of Topical l‐Glutamine on Oral Mucositis in 5‐Fluorouracil‐Treated Mice
Source: J Oral Pathol Med. 2026 Apr 7;55(6):705–17. doi: 10.1111/jop.70135 (PMC13333535; doi:10.1111/jop.70135)
Supplement: Supplementary file 2 — Table S1: Effects of 5‐FU in blood Leukocytes number. Table S2: Effects of 5‐FU in body weight. Table S3: Lesion area. Table S4: Necrotic area. Table S5:. Histopathological score (HE). Table S6: Effects of l‐Glutamine on NAG activity. Table S7: Effects of L‐Glutamine on TNF‐α production. Table S8: Effects of L‐Glutamine on the mast cell number. Table S9: Effects of L‐Glutamine on collagen fiber quantification. Table S10: Effects of L‐Glutamine on TGF‐β1 levels. Table S11: Effect of L‐Glutamine on the proliferative marker ki67. [file JOP-55-705-s001.docx]

**DATA MANUSCRIPT**

**Table 1.** Effects of 5-FU in blood Leukocytes number.

|  | Blood Leukocytes (µl^3^/blood) |
| --- | --- |
| **Before 5-FU** | 3550 |
|  | 5000 |
|  | 3450 |
|  | 6150 |
|  | 5950 |
|  | 5000 |
|  | 6690 |
|  | 2850 |
| **Post-5FU** |  |
|  | 1830 |
|  | 1650 |
|  | 2300 |
|  | 1300 |
|  | 1700 |
|  | 3650 |
|  | 2100 |
|  | 2700 |

Student t-test.** p 0.004 n= 8 animals.

An paired t test showed a significant difference between groups (t=4.203) df=7, p = 0.004 The mean difference between groups was −2676 ± 636 leukocytes (95% CI −4182 to −1171). The effect size was = 0.716).

**Table 2.** Effects of 5-FU in body weight.

|  | H_2_O + 5FU | | | | | | | | | |
| --- | --- | --- | --- | --- | --- | --- | --- | --- | --- | --- |
| Days post implantation | Weight (g) | | | | | | | | | |
|  |  | Mean |  | SD |  |  |  |  |  |  |
| 1 |  | 17.7 |  | 1.801137 |  |  |  |  |  |  |
| 3 |  | 14.9 |  | 1.275006 |  |  |  |  |  |  |
| 6 |  | 12.9 |  | 1.155662 |  |  |  |  |  |  |
|  | L-glutamine+ 5FU | | | | | | | | | |
| 1 |  | 17.3 |  | 1.852649 |  |  |  |  |  |  |
| 3 |  | 15.0 |  | 1.604184 |  |  |  |  |  |  |
| 6 |  | 14.5 |  | 0.931722 |  |  |  |  |  |  |

ANOVA 2Way showed a significant difference between groups at 6 days.

Day 1 = (t(0.7342) df = 69). The mean difference between groups was 0.4133, (95% CI −-0.9643 to 1.791). p= 0.734;

Day 3 = (t(0.2021) df = 69). The mean difference between groups was 0.4133, (95% CI –1.638 to 1.388). p= 0.202;

Day 6= (t(2.454) df = 69). The mean difference between groups was 0.4133, (95% CI –3.179 to -0.0043). p= 0.049;

**Table 3.** Lesion area

|  | Lesion area mm ^2^ |
| --- | --- |
| **CTL- 5FU** | 3.406 |
|  | 4.656 |
|  | 2.986 |
|  | 3.252 |
|  | 4.479 |
| **GLU-5-FU** | 2.015 |
|  | 1.470 |
|  | 3.410 |
|  | 1.724 |
|  | 1.662 |

Student t-test.** p= 0.0082. n= 5 animals.

An unpaired t test showed a significant difference between groups (t(3.490) df = 8). The mean difference between groups was −1.700 ± 0.4870 (95% CI −2.823 to −0.5768). R squared =0.6036

**Table 4.** Necrotic area

|  | Necrotic area mm ^2^ |
| --- | --- |
| **CTL- 5FU** | 4.22  1.48  3.92  2.29  1.85 |
| **GLUT- 5FU** | 1.52  0.10  1.82  0.67  0.05 |

* p = Student t-test.0.0202 n= 5 animals.

An unpaired t test showed a significant difference between groups (t) = 2.892, p = 0.0202. The mean difference between groups was −1.918 ± 0.6634 (95% CI −3.448 to −0.3885). R squared 0.5110.

**Table 5**. Histopathological score (HE)

| Score | Epithelial alterations: hyperkeratosis and hyperplasia =1 point; presence of ulceration = 2 points; presence of both ulceration and proliferative alterations =3 points.  Inflammatory alterations = Inflammatory cells (0-3), edema (0-3), hemorrhage (0-3) |  |
| --- | --- | --- |
| CTL- 5FU | 9  7  9  11  9 |  |
| GLUT- 5FU |  |  |
|  | 2 |  |
|  | 5 |  |
|  | 7 |  |
|  | 9 |  |
|  | 9 |  |
|  |  |  |

Student t-test.* p= 0.028. n= 5 animals.

An unpaired t test showed a significant difference between groups (t) = 2.661, df=8. The mean difference between groups was -3.800 ± 1.428 (95% CI −7.094 to −0.5064). R squared 0.4694.

Effects of L-Glutamine on MPO activity

|  | MPO activity (O.D./g tissue) |
| --- | --- |
| CTL- 5FU | 2.7544  4.1263  11.8449  2.2770  10.903  12.162  13.507 |
| GLUT- 5FU | 2.8887 |
|  | 5.1554 |
|  | 7.1958 |
|  | 2.3596 |
|  | 1.2865 |
|  | 1.9335 |
|  | 2.8390 |

Student t-test.* p= 0.0337 n= 7 animals.

An unpaired t test showed a significant difference between groups (t) = 2.397, df=12. The mean difference between groups was −4.845 ± 2.021 (95% CI −9.249 to −0.4419). R squared 0.3239.

**Table 6.** Effects of L-Glutamine on NAG activity

|  | NAG activity (nmol/g wet tissue) |
| --- | --- |
| **CTL- 5FU** | 14.5747  19.3739  10.3928  10.6378  10.1693  12.0330  9.01497 |
| **GLUT- 5FU** | 11.9266 |
|  | 7.64299 |
|  | 6.81528 |
|  | 8.34516 |
|  | 8.67747 |
|  | 10.8944 |
|  | 5.25763 |

Student t-test.*p= 0.0357. n= 7 animals.

An unpaired t test showed a significant difference between groups (t) = 2.366, df=12. The mean difference between groups was −3.805 ± 1.608 (95% CI −7.309 to −0.3012). R squared 0.3181.

**Table 7.** Effects of L-Glutamine on TNF-α production

|  | TNF-α (pg/mg wet tissue) |
| --- | --- |
| **CTL- 5FU** | \| 2.6637 \| \| --- \| \| 2.2422 \| \| 2.6058 \| \| 4.3557 \| \| 3.6899 \| \| 4.9543 \| \| 3.9894 \| \| 2.6637 \| |
| **GLUT- 5FU** | 2.6618 |
|  | 0.1839 |
|  | *5.2859** |
|  | 1.9149   \| 0.7787 \| \| --- \| \| 1.4424 \| \| 1.9735 \| |

Student t-test.* p 0.0037 n= 7 animals. Outliers are indicated in blue

An unpaired t test showed a significant difference between groups (t) = 3.757, df=10. The mean difference between groups was −2.206 ± -0.8979 (95% CI −3.525 to −0.8979). R squared 0.5853.

**Table 8.** Effects of L-Glutamine on the mast cell number

|  | Mast cell number/ slide |
| --- | --- |
| **CTL- 5FU** | \| 8.7143 \| \| --- \| \|  \| \| 15.8345 \| \| 24.7143 \| \| 17.2857 \| \| 11.8333 \| |
| **GLUT- 5FU** | 5.8182 |
|  | 7.8571 |
|  | 7.1667 |
|  | 6.4286 |
|  | 5.4286 |

Student t-test.* p= 0.0106, n= 5 animals.

An unpaired t test showed a significant difference between groups (t) = 3.318, df=8. The mean difference between groups was −9.130 ± 2.752 (95% CI −15.47 to −2.785). R squared 0.5791.

**Table 9.** Effects of L-Glutamine on collagen fiber quantification

|  | Collagen fiber µm^2^ |
| --- | --- |
| **CTL- 5FU** | \| 29677.002 \| \| --- \| \| 35551.761 \| \| 23240.146 \| \| 35859.705 \| \| 30200.103 \| |
| **GLUT- 5FU** | 45977.358 |
|  | *27543.909** |
|  | 40309.002 |
|  | 47823.028 |
|  | 43000.354 |

* Unpaired t test p 0.0029, n= 5 animals. Outliers are indicated in blue

An unpaired two-tailed t test showed a significant difference between groups (t) = 4.470, df=7. The mean difference between groups was 13412 ± 3001 (95% CI −6316 to 20507). R squared 0.7405.

**Table 10.** Effects of L-Glutamine on TGF-β1 levels

|  | TGF-β1 levels (pg/mg wet tissue) | |
| --- | --- | --- |
| **CTL- 5FU** | \| 9.324709302 \| \| --- \| \| 11.9724186 \| \|  \| \| 7.178208773 \| \| 6.582620321 \| \| 8.243079026 \| \| 4.82019544 \| \| 5.334427669 \| | |
| **GLUT- 5FU** | 6.691753063 | |
|  | 10.06034431 | |
|  | 7.733705613 | |
|  | 4.373713786 | |
|  | 5.60093423  11.77880934 |  |
|  | 11.0232234 |  |
|  |  | |

Unpaired t test p 0.7084, n= 7 animals.

An unpaired two-tailed t test showed a no difference between groups (t) = 0.3830, df=12. The mean difference between groups was −0.5438 ±1.420 (95% CI −2.550 to −3.638). R squared 0.01207.

**Table 11**.Effect of L-Glutamine on the proliferative marker ki67

|  | Cell marker Ki67/ slide |
| --- | --- |
| **CTL- 5FU** | 1260  1650  1730 |
| **GLUT- 5FU** | 706  980  1212 |

* Unpaired t test p 0.0479. n= 3 animals

An unpaired two-tailed t test showed a significant difference between groups (t) = 2.818, df=4. The mean difference between groups was −580,7 ± 206,1 (95% CI −1153 to −8.536). R squared 0.6650.
